# P-761. Advancing TB Patient Care: Utilizing XPERT MTB/XDR for Additional Drug Resistance Detection in an Indian Tertiary Care Context

**DOI:** 10.1093/ofid/ofae631.956

**Published:** 2025-01-29

**Authors:** Haripriya Reddy challa, Bhuvaneswari Ventrapragada, Ankit Mittal, B Harshith

**Affiliations:** AIG hospitals, hyderabad, Telangana, India; AIG hospitals, hyderabad, Telangana, India; AIG Hospitals, Hyderabad, Uttar Pradesh, India; AIG hospitals, hyderabad, Telangana, India

## Abstract

**Background:**

The World Health Organization (WHO) underscores the importance of universal access to drug susceptibility testing (DST) as a part of its END TB strategy. Rapid molecular tests with shorter turnaround time (TAT) are indispensable in this endeavor. Xpert MTB/XDR is one such test which identifies Mycobacterium tuberculosis complex (MTBC) and resistance to isoniazid, fluoroquinolones, aminoglycosides, and ethionamide. In this study we looked at clinical utility of this test in real world

**Figure:**
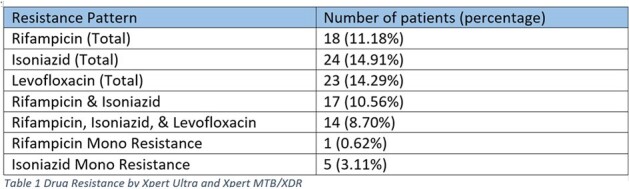

**Methods:**

Data of patients on whom Xpert MTB/XDR was done from September 2022 to April 2024 was collected retrospectively and analysed to see additional drug resistance capture. For baseline drug resistance data, we collected data of patients before September 2022 on whom additional DST (other than Rifampicin resistance testing by GeneXpert), including Line Probe Assays (LPA) and phenotypic DST (pDST) on culture isolates was done.

**Results:**

Baseline drug susceptibility data were available for 39 patients, comprising 19 patients with first-line LPA, 14 with both first and second-line LPA, and 15 with only pDST. Among them, 3/39(7.69) exhibited rifampicin resistance, 7/39 (17.9) isoniazid resistance, 3/39(7.69) quinolone resistance, and 4/39(10.2) aminoglycoside resistance. Xpert MTB/XDR was performed on 193 patients, with 161 testing positive for MTBC. The median age of the study population was 64.5 years (IQR 29.7-56), with 64.7% being male. Rifampicin resistance was detected in 18/161 (11.18%) cases, isoniazid resistance in 24/161 (14.9%), fluoroquinolone resistance in 23/161 (14.29%), and aminoglycoside resistance in 4/161 (2.4%).Among all Rifampicin resistance patients 94% (14/18) are quinolone resistance. Isoniazid monoresistance is only 3% (5/161).

**Conclusion:**

In clinical practice, compliance to FL-LPA, SL-LPA and pDST is low because of higher cost and TAT effecting capture of additional drug resistance especially of quinolones, which is an integral part of the drug resistant TB regimen. This is also important in special population like chronic liver disease or drug induced liver injury where pyrazinamide is replaced by quinolones. Xpert MTB/XDR is an efficient and cost-effective alternative to traditional DST methods with a lower TAT and should be integrated in National TB program

**Disclosures:**

**All Authors**: No reported disclosures

